# Physiological Effects of Visual Stimulation with Forest Imagery

**DOI:** 10.3390/ijerph15020213

**Published:** 2018-01-26

**Authors:** Chorong Song, Harumi Ikei, Yoshifumi Miyazaki

**Affiliations:** 1 Center for Environment, Health and Field Sciences, Chiba University, 6-2-1 Kashiwa-no-ha, Kashiwa, Chiba 277-0882, Japan; crsong1028@chiba-u.jp (C.S.); ikei0224@ffpri.affrc.go.jp (H.I.); 2 Department of Wood Engineering, Forestry and Forest Products Research Institute, 1 Matsunosato, Tsukuba, Ibaraki 305-8687, Japan

**Keywords:** forest therapy, shinrin-yoku, forest imagery, autonomic nervous activity, prefrontal cortex activity, heart rate variability, near-infrared spectroscopy, semantic differential method, physiological relaxation, preventive medical effect

## Abstract

This study was aimed to clarify the physiological effects of visual stimulation using forest imagery on activity of the brain and autonomic nervous system. Seventeen female university students (mean age, 21.1 ± 1.0 years) participated in the study. As an indicator of brain activity, oxyhemoglobin (oxy-Hb) concentrations were measured in the left and right prefrontal cortex using near-infrared time-resolved spectroscopy. Heart rate variability (HRV) was used as an indicator of autonomic nervous activity. The high-frequency (HF) component of HRV, which reflected parasympathetic nervous activity, and the ratio of low-frequency (LF) and high-frequency components (LF/HF), which reflected sympathetic nervous activity, were measured. Forest and city (control) images were used as visual stimuli using a large plasma display window. After sitting at rest viewing a gray background for 60 s, participants viewed two images for 90 s. During rest and visual stimulation, HRV and oxy-Hb concentration in the prefrontal cortex were continuously measured. Immediately thereafter, subjective evaluation of feelings was performed using a modified semantic differential (SD) method. The results showed that visual stimulation with forest imagery induced (1) a significant decrease in oxy-Hb concentrations in the right prefrontal cortex and (2) a significant increase in perceptions of feeling “comfortable,” “relaxed,” and “natural.”

## 1. Introduction

More than half of the global population currently live in urban environments [1], and 66% of individuals are expected to live in urban areas by the year 2050 [2]. Although urbanization has led to improvements in many areas such as housing, employment, education, equality, quality of living environment, social support, and health services [3], changes occurring over a very short period have been very drastic from an evolutionary perspective. The gap between the highly urbanized and artificial environment that we modern humans currently inhabit, and our physiological functions which are best adapted to natural settings, is likely to contribute to a state of stress among people. Recent research has shown that city dwellers are constantly exposed to stressors and that urban living is associated with an increased risk of health problems [4,5,6,7].

As a result of such stressful situations in modern society, effective methods for coping with stress and for relaxation are receiving increasing attention. One such method is interaction with nature, because nature-based experiences are known to have a relaxing effect. Recent research has demonstrated that the natural environment plays an important role in health promotion and that there is a positive relationship between nature-derived stimuli and human health [8,9,10,11,12].

In particular, there has been considerable and increasing attention in using the forest environment as a place for recreation and health promotion. This approach is called “Shinrin-yoku” in Japan and means “taking in the forest atmosphere through all of our senses” [13]. It suggests “forest bathing,” which is a health promotion method that uses proven effects of a forest environment (such as relaxation) that can improve health of the body and mind. Based on the results of studies such as the above, the idea of “forest therapy” has been proposed. The evidence-based practice of “forest bathing (shinrin-yoku)” seeks to achieve preventive medical effects via the improvement of weakened immune functions and the prevention of diseases by achieving a state of physiological relaxation through exposure to forest-origin stimuli. Many studies have demonstrated the effects of walking in and/or viewing forests in mitigating stress states and inducing physiological relaxation [14,15,16,17,18,19,20,21]. Spending time in forests improves immune functions [22,23], and these effects last for approximately one month [24]. In addition, experiments with adults who are at risk of stress- and lifestyle-related diseases such as high blood pressure, diabetes, and depression, as well as elderly individuals, have found that various activities performed in forests have positive effects [25,26,27,28,29,30,31,32]. Restorative effects of forest environments on psychological stressors or mental fatigue, including decreased depressive symptoms and improved mood states, have been reported [18,20,33,34].

In addition, studies have been aimed at identifying elements of forests that bring about the above benefits. Forest-based stimuli are intuitively perceived through the five senses. Of the five senses, physiological effects of olfactory stimulation due to aromatic volatile substances derived from trees called phytoncides have been characterized in detail. The earliest experimental report in laboratory experiment, published in 1992 [35], showed that olfactory stimulation with Taiwan cypress essential oil significantly decreased blood pressure. Subsequently, other studies using Hinoki cypress (*Chamaecyparis obtusa*), a coniferous tree, have been conducted, clearly showing that inhalation of its leaf oil induced reduction in oxyhemoglobin concentrations in the prefrontal cortex and increased parasympathetic nervous activity, known to be associated with a relaxed state [36]. Wood oil from the above tree was found to increase natural killer cell activity and improve immune functions [37]. In addition, it has been reported that inhalation of α-pinene and d-limonene, which are major components of forest odor, decreased systolic blood pressure [38], enhanced parasympathetic nervous activity, and decreased heart rate [39,40].

However, most previous studies of physiological effects of forest-origin stimuli on humans have used olfactory stimulation, and examination of visual, auditory, and tactile stimuli have been limited. In contrast, recent developments in image projection technology have enabled visualization of images such as forests with greater clarity. Because visual stimulation derived from forests is expected to be familiar and convenient, we focused on visual stimulation. Ulrich et al. [41] investigated the effects of viewing a video about a natural environment on stress states, compared with effects of a video about an urban environment. Scenes of forests and streams were used to represent a natural environment. Measured parameters included skin conduction, muscle tension, and heart rate. The results showed that stress states were rapidly reduced upon exposure to the natural environment, compared with exposure to the urban environment. This was a pioneering study on the physiological effects of visual stimulation with forest imagery on humans; however, the study was limited by the fact that they only used indices of autonomic nervous activity as physiological responses.

In this study, we investigated the physiological effects of visual stimulation with forest imagery on left and right prefrontal cortex activity assessed using near-infrared time-resolved spectroscopy and on autonomic nervous activity assessed using heart rate variability.

## 2. Materials and Methods

### 2.1. Participants

Seventeen female Japanese university students (mean age ± standard deviation: 21.1 ± 1.0 years) participated in the study. Mean height and weight were 158.2 ± 4.5 cm and 51.9 ± 6.2 kg, respectively. Participants who smoked, those currently in treatment for any disease, and those in their menstrual period during the study were excluded. All participants were informed about the aims and procedures of the study. After receiving a description of the experiment, they gave written consent to participate in the study. The study was conducted in accordance with guidelines of the Declaration of Helsinki, and the protocol was approved by the Ethics Committees of the Center for Environment, Health and Field Sciences, Chiba University, Japan (project identification no. 5).

### 2.2. Visual Stimulation

**Figure 1:**
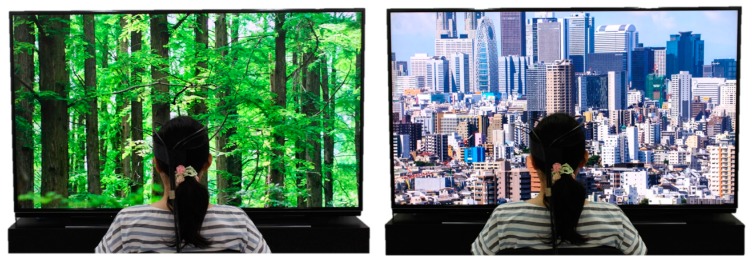
The scene during visual stimulation: (**left**) forest image; (**right**) city image.

Visual stimulation was conducted using a 4K-compatible high vision liquid crystal display television of width 1872 mm and height 1053 mm and 3840 × 2160 pixel resolution (85V type, TH-85AX900 by Panasonic, Osaka, Japan). A forest landscape showing Metasequoia (*Metasequoia glyptostroboides*) was used as forest imagery, and a scene of Shinjuku in Tokyo was used as control city imagery (Figure 1). The display was 1.4 m away from the participants.

### 2.3. Study Protocol

**Figure 2:**
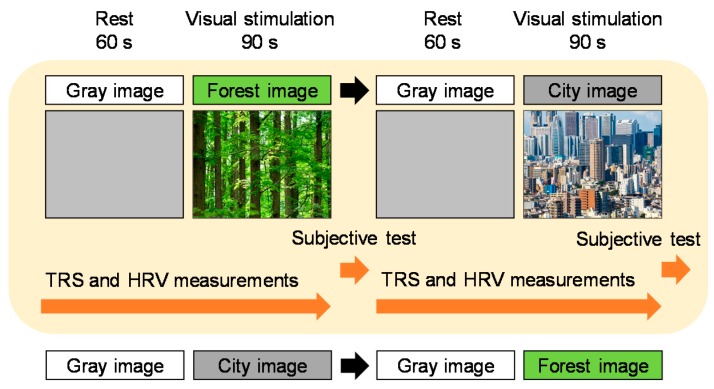
Study protocol. TRS: near-infrared time-resolved spectroscopy; HRV: heart rate variability.

Physiological measurements were performed in a chamber with an artificial climate in the Center for Environment, Health and Field Sciences, Chiba University. This chamber was maintained at 24 °C, 50% relative humidity, and 50 lux illumination. The participants received a description of the experiment in a waiting room, and then moved into the chamber with an artificial climate. After sensors for physiological measurement were fitted, participants received a description of the measurement procedure while sitting. The study protocol is shown in Figure 2. After resting while viewing a gray background for 60 s (rest period), each participant was separately exposed to forest and city (control) images for 90 s each, while maintaining a sitting position. During the testing procedure, measurements of participants’ physiological responses were continually performed. After finishing the 90 s visual stimulation, subjective evaluations were conducted. To eliminate any influences due to the order of viewing forest and city images, visual stimuli were presented in a counterbalanced order.

### 2.4. Physiological Measurement

#### 2.4.1. Near-Infrared Time-Resolved Spectroscopy (TRS)

**Figure 3:**
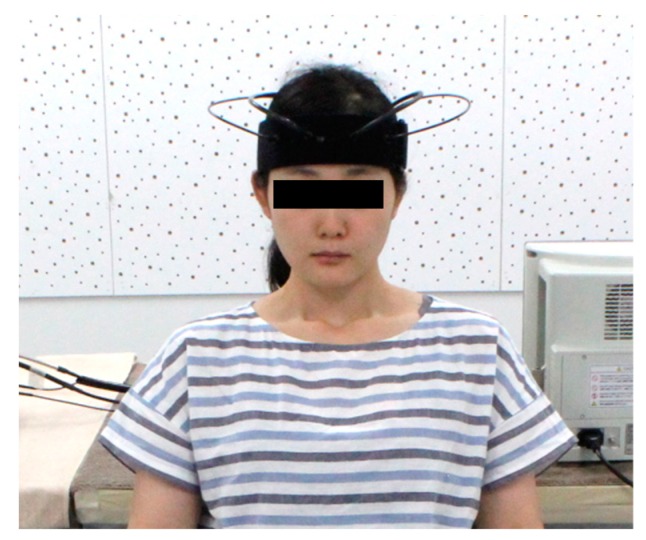
Participant undergoing near-infrared time-resolved spectroscopy measurement.

As an indicator of brain activity, TRS, a near-infrared spectroscopy (NIRS)-based method, was used [42,43]. The sensors were mounted at approximately Fp1 and Fp2 of the international 10–20 system (electroencephalogram) on the participant’s forehead (Figure 3), and oxyhemoglobin (oxy-Hb) concentration in the prefrontal cortex measured (TRS-20 system; Hamamatsu Photonics K.K., Shizuoka, Japan). Principles of the NIRS measurement are as follows. With an increase in local brain activity, brain blood flow increases and leads to significant perfusion such that quantity of brain blood flow exceeds oxygen consumption [44]. Consequently, oxy-Hb increases, and this increase can be detected. In addition, it is known that increases or decreases in quantity of blood flow in the brain are consistent with those of oxy-Hb [45], and it is thought that a decrease in oxy-Hb concentration causes physiological calming. The oxyhemoglobin (oxy-Hb) concentrations in the left and right prefrontal cortex were measured during the 60 s rest period and the 90 s visual stimulation period. Data measured using TRS-20 differ in sampling time. In the present experiment, data were measured at approximately 1.0–1.2 s intervals. We transformed the data using linear interpolation every second in order to show time series data of oxy-Hb concentration in the left and right prefrontal cortex. Each data point was calculated as the difference from the average of the 60 s rest period. Changes every 30 s and the overall mean during the 90 s visual stimulation period were analyzed.

#### 2.4.2. Heart Rate Variability (HRV) and Heart Rate

As indicators of autonomic nervous activity, HRV and heart rate were used [46,47]. HRV was analyzed for the periods between consecutive R waves in the electrocardiogram (RR intervals) as measured by a portable electrocardiogram (Activtracer AC-301A; GMS, Tokyo, Japan). The power levels of the low-frequency (LF: 0.04–0.15 Hz) and high-frequency (HF: 0.15–0.40 Hz) components of HRV were calculated using the maximum-entropy method (MemCalc/Win; GMS, Tokyo, Japan) [48]. The HF power reflects parasympathetic nervous activity. The LF/HF ratio reflects sympathetic nervous activity. Changes in values of HF and LF/HF every 30 s period and overall mean during the 90 s of visual stimulation period were acquired, respectively. All data were calculated as differences from averages of the 60 s rest period.

### 2.5. Psychological Measurement

Psychological measurements were performed using the modified semantic differential (SD) method [49]. The SD method tests subjective evaluations of participants through a questionnaire with opposing adjectives, each of which was evaluated on a 13-point scale. Three pairs of adjectives were assessed; “comfortable–uncomfortable,” “natural–artificial,” and “relaxed–awakening.”

### 2.6. Statistical Analysis

The software Statistical Package for Social Sciences (v21.0, IBM Corp., Armonk, NY, USA) was used for all statistical analyses.

Changes in physiological indices every 30 s were analyzed using paired *t*-tests with Holm correction to compare physiological responses between forest and city images; thus, the Holm correction [50,51] was applied three times. The Holm correction procedure is as follows. First, all *p*-values are sorted by size and then compared with increasing limits. The lowest limit is the overall limit divided by three, and the smallest *p*-value is to be compared with 0.05/3 or ca. 0.017. If the smallest *p*-value is >0.017, the process stops, but if it is smaller, the next smallest *p*-value is divided by two (*p* = 0.025). The process continues in a similar manner in that if the *p*-value is significant, the next smallest value is divided by one (*p* = 0.050). Overall mean values over the 90 s visual stimulation were analyzed using paired *t*-tests to compare physiological responses between the images.

Wilcoxon signed-rank test was used to analyze differences in psychological indices between the images.

One-sided tests were used for both comparisons, because we hypothesized that humans would be more relaxed after viewing forest imagery than city imagery.

## 3. Results

### 3.1. Physiological Effects

#### 3.1.1. TRS

**Figure 4:**
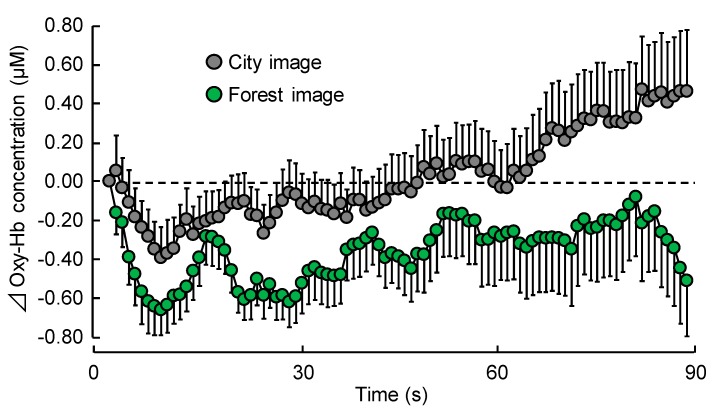
Change in oxyhemoglobin (oxy-Hb) concentration in the right prefrontal cortex during visual stimulation with forest and city images, every second over a 90 s period. Data are expressed as mean ± standard error, *n* = 17.

Figure 4 shows time-dependent oxy-Hb concentration change every second in the right prefrontal cortex during visual stimulation with forest or city images. Mean baseline of 60 s oxy-Hb concentration during the rest period did not significantly differ between the two stimuli (forest: 43.67 ± 1.23 μM (mean ± standard error), city: 43.58 ± 1.22 μM; *p* > 0.05). However, oxy-Hb concentrations decreased immediately after viewing forest imagery and remained lower than the initial value throughout the rest of the experiment. Although oxy-Hb concentrations also decreased immediately after viewing city imagery, the value gradually increased during the course of the experiment.

**Figure 5:**
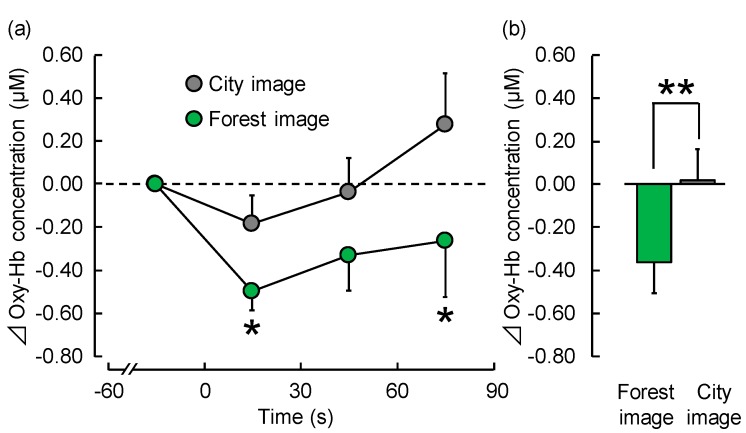
Thirty-second average and overall mean oxyhemoglobin (oxy-Hb) concentration in the right prefrontal cortex during visual stimulation with forest and city images. (**a**) Changes in each 30 s average oxy-Hb concentration over 90 s. (**b**) Overall mean oxy-Hb concentrations. Data are expressed as means ± standard errors, *n* = 17, * *p* < 0.05, ** *p* < 0.01, as determined by the paired *t*-test (one-sided); the Holm correction was applied.

Each 30 s average of oxy-Hb concentration in the right prefrontal cortex during visual stimulation with forest and city images is shown in Figure 5a. Mean oxy-Hb concentration in the 1–30 s period was −0.50 ± 0.09 μM during exposure to forest imagery, and −0.19 ± 0.13 μM during exposure to city imagery, showing a significant decrease (*p* < 0.05). Even in the 61–90 s period, it showed a significant decrease (*p* < 0.05) with mean concentration being −0.26 ± 0.26 μM during exposure to forest imagery and 0.27 ± 0.24 μM during exposure to city imagery. However, there was no significant difference in mean oxy-Hb concentrations in the 31–60 s period (forest: −0.33 ± 0.16 μM; city: −0.04 ± 0.16 μM).

Figure 5b shows overall mean oxyhemoglobin (oxy-Hb) concentration in the right prefrontal cortex during visual stimulation with forest and city images. Viewing forest imagery significantly increased oxy-Hb concentration compared with viewing city imagery (forest: −0.36 ± 0.14 μM; city: 0.02 ± 0.14 μM; *p* < 0.05). 

However, there was no significant difference in the left prefrontal cortex (forest: −0.20 ± 0.16 μM; city: 0.05 ± 0.18 μM; *p* > 0.05).

#### 3.1.2. HRV and Heart Rate

There were no significant differences in the HF value, which is an index of parasympathetic nervous activity, LF/HF ratio, which is an index of sympathetic nervous activity, and heart rate between participants viewing forest and city images.

### 3.2. Psychological Effects

**Figure 6:**
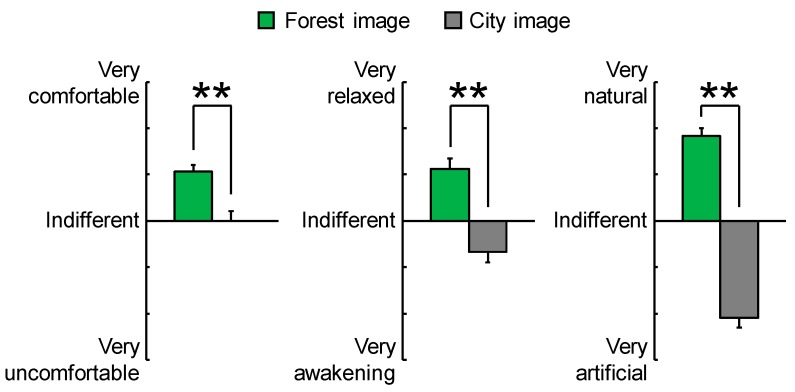
Subjective feelings measured with the modified semantic differential method after viewing forest and city images. Data are expressed as means ± standard errors, *n* = 17, ** *p* < 0.01 as determined by the Wilcoxon signed-rank test.

Figure 6 shows the results of subjective feelings as measured by a modified SD questionnaire after visual stimulation with forest and city images. Participants felt more “comfortable,” “natural,” and “relaxed” when they viewed forest imagery, compared with city imagery (*p* < 0.01).

## 4. Discussion

This study examined the physiological effects of visual stimulation with forest imagery on left and right prefrontal cortex activity assessed using TRS and on autonomic nervous activity assessed using HRV. In addition, subjective assessments of psychological relaxation were conducted. Results of physiological measurements showed that viewing forest imagery significantly decreased oxy-Hb concentrations in the right prefrontal cortex, compared with viewing city imagery. Table 1 shows the results of physiological and psychological measurements.

Assessing the effects of spending time in a forest environment on brain activity, Park et al. [14] reported that walking for 15 min in a forest area reduced total hemoglobin concentration in the left prefrontal cortex, compared with walking in a city area. Joung et al. [52] found that viewing a forest landscape, compared to viewing an urban landscape, from the roof of an urban building for 15 min decreased total hemoglobin concentration and oxy-hemoglobin concentration in the prefrontal cortex. Testing the effect of olfactory stimulation by forest-origin stimuli on brain activity, Ikei et al. [36] demonstrated that inhalation of Hinoki cypress leaf oil reduced oxyhemoglobin concentrations in the right prefrontal cortex. However, studies of the effects of forest stimuli on brain activity are limited. The results of this study show a significant decrease in oxy-Hb concentrations in the right prefrontal cortex, partly consistent with findings of previous studies in the context of calming brain activity. However, reason(s) for no significant difference in the left prefrontal cortex is unknown; it is necessary to obtain more data on the influence of forest-derived stimuli on prefrontal cortex activity to clarify any differences in response between left and right prefrontal cortices. Autonomic nervous activity (heart rate, sympathetic nervous activity, and parasympathetic nervous activity) did not change significantly. Kahn et al. [53] also investigated the physiological effect of plasma display windows using heart rate. In an office setting, 90 participants (30 per group) were exposed either to (a) a glass window that afforded a view of a nature scene, (b) a plasma window that afforded a real-time HDTV view of essentially the same scene, or (c) a blank wall. Results showed that in terms of heart rate recovery from low-level stress, the glass window was more restorative than a blank wall. However, the plasma window was no different from the blank wall. A similar result was obtained with respect to heart rate. These findings need to be confirmed using different experimental design settings and visual stimulation methods in future studies.

In addition, results of subjective assessments of psychological relaxation showed that viewing forest imagery significantly increased perceptions of feeling “comfortable,” “relaxed,” and “natural” compared with viewing city imagery. These psychological benefits of viewing forest imagery can be considered significant in modern times, given that mental health problems associated with living in urban environments are profound [5,7]. Further, application of these findings is expected to play an important role in improvement of psychological stress states in the future.

The findings of this study provide rational scientific evidence for beneficial physiological and psychological effects of viewing forest imagery in young women in their 20s. To generalize these findings, further studies based on a larger sample including other demographic groups such as males and different age groups, are required. Further, it is necessary to examine such effects not only in healthy individuals but also in populations who experience a heightened state of stress in daily life. In addition, in future studies, it will be necessary to elucidate the effect of differences in magnification, angles, and display hue between forest and city imagery.

## 5. Conclusions

The findings of this study provide significant scientific evidence of the physiological effects of visual stimulation with forest imagery on brain activity and autonomic nervous activity. Viewing forest imagery induced (1) a significant decrease in oxy-Hb concentrations in the right prefrontal cortex and (2) a significant increase in perceptions of feeling “comfortable,” “relaxed,” and “natural.” These findings indicate that viewing forest imagery may induce physiological and psychological relaxation.

## Figures and Tables

**Table 1:** Physiological and psychological measurements.

Physiological Measurement (*n* = 17)	Forest Image	City Image	*p*-Value	Significance Level
	Mean ± SE	Mean ± SE		
	µM	µM		
Oxy-Hb concentration in the right prefrontal cortex				
1–30 s	−0.50 ± 0.09	−0.19 ± 0.13	0.024	* *p* < 0.05
31–60 s	−0.33 ± 0.16	−0.04 ± 0.16	0.097	NS
61–90 s	−0.26 ± 0.26	0.27 ± 0.24	0.011	* *p* < 0.05
Overall mean	−0.36 ± 0.14	0.02 ± 0.14	0.006	** *p* < 0.01
Oxy-Hb concentration in the left prefrontal cortex				
1–30 s	−0.31 ± 0.09	−0.13 ± 0.14	0.131	NS
31–60 s	−0.26 ± 0.16	−0.10 ± 0.18	0.260	NS
61–90 s	−0.02 ± 0.27	0.37 ± 0.30	0.087	NS
Overall mean	−0.20 ± 0.16	0.05 ± 0.18	0.112	NS
**Psychological Measurement (*n* = 17)**	**Forest Image**	**City Image**	***p*Value**	**Significance Level**
	**Mean ± SE**	**Mean ± SE**		
	**Score**	**Score**		
“Comfortable” feeling	2.1 ± 0.3	0.0 ± 0.4	<0.001	** *p* < 0.01
“Relaxed” feeling	2.2 ± 0.4	−1.4 ± 0.4	<0.001	** *p* < 0.01
“Natural” feeling	3.6 ± 0.3	−4.2 ± 0.4	<0.001	** *p* < 0.01

* *p* < 0.05, ** *p* < 0.01, NS: not significant.
